# Measurement-Based Mental Health Care for Adolescents in Africa: Protocol for a Computerized Adaptive Test

**DOI:** 10.2196/98369

**Published:** 2026-09-08

**Authors:** Bianca Moffett, Mary Bitta, Darryn Williams, Jonathan Doenz, Holly Erskine, Constance Mabia, Tamera Nkuna, Stash Gomes, Alastair van Heerden, Marguerite Marlow, Joemer Maravilla, Mark Tomlinson, Michelle Craske, Mary-Anne Hartley, Robert Gibbons

**Affiliations:** 1 SAMRC/Wits-Agincourt, School of Public Health, Faculty of Health Sciences, University of the Witwatersrand, 27 St Andrew's Road, Parktown, Johannesburg, Gauteng, 2193, South Africa, 27 117172085; 2 Brain and Mind Institute, Aga Khan University Nairobi, Nairobi, Nairobi County, Kenya; 3 Harvard Medical School, Department of Global Health and Social Medicine, Harvard University, Boston, MA, United States; 4 Department of Psychiatry, University of Oxford, Oxford, England, United Kingdom; 5 Laboratory for Intelligent Global Health and Humanitarian Response Technologies, École Polytechnique Fédérale de Lausanne, Lausanne, Vaud, Switzerland; 6 School of Public Health, The University of Queensland, Brisbane, Queensland, Australia; 7 Queensland Centre for Mental Health ResearchWacol, Queensland, Australia; 8 University of Washington, Institute for Health Metrics and Evaluation, Seattle, WA, United States; 9 Aves Mental Health, Nairobi, Kenya; 10 School of Clinical Medicine, Faculty of Health Sciences, University of the Witwatersrand, Johannesburg, Gauteng, South Africa; 11 Institute for Life Course Health Research, Faculty of Medicine and Health Sciences, Stellenbosch University, Stellenbosch, Western Cape, South Africa; 12 Institute of Health Sciences and Nursing, Far Eastern University, Manilla, National Capital Region, Philippines; 13 School of Nursing and Midwifery, Queen's University Belfast, Belfast, Northern Ireland, United Kingdom; 14 Department of Psychology and Department of Psychiatry and Biobehavioral Sciences, University of California, Los Angeles, Los Angeles, CA, United States; 15 Department of Medicine and Department of Public Health Sciences (Biostatistics), University of Chicago, Chicago, IL, United States

**Keywords:** measurement-based care, adolescent mental health, computerized adaptive testing, depression, anxiety, Africa, clinical decision support

## Abstract

**Background:**

Adolescent mental health care is constrained not only by limited treatment capacity but also by the absence of precise and efficient tools to identify and triage those in need. Measurement-based mental health care relies on standardized assessments to guide identification, treatment selection, and ongoing monitoring of mental disorders. However, conventional assessments present a trade-off: diagnostic interviews lack feasibility at scale, while brief screening tools result in misclassification and suboptimal allocation of limited mental health resources. Computerized adaptive testing addresses this trade-off by dynamically tailoring assessments to individuals, maximizing measurement precision while minimizing assessment burden. Despite these advances, no adaptive assessment for adolescent mental health has been developed or calibrated using African data.

**Objective:**

The AfriCAT study aims to develop and internally validate a precision computerized adaptive assessment of adolescent depression and anxiety using nationally representative Kenyan data, with the longer-term goal of supporting measurement-based mental health care across diverse African contexts.

**Methods:**

AfriCAT is a mixed methods study integrating psychometric modeling, adaptive simulation, modular clinical decision support networks, participatory workshops, discrete choice experiments (DCEs), and stakeholder interviews. *Diagnostic Interview Schedule for Children, version 5* (*DISC-5*) modules assessing major depressive disorder, generalized anxiety disorder, social phobia, and posttraumatic stress disorder among adolescents aged 10 to 17 years from the Kenya National Adolescent Mental Health Survey (N=5155) will form the item bank. Adaptive engines will be developed using a bifactor multidimensional item response theory framework and a modular neural architecture. Simulation-based internal validation will evaluate precision, efficiency, and diagnostic classification relative to *DISC-5* diagnoses. Participatory workshops in Kenya and South Africa, DCEs to quantify adolescent preferences, and stakeholder interviews will inform refinement of adolescent- and provider-facing prototypes.

**Results:**

Ethical approval has been obtained from the Human Research Ethics Committee (Medical) at the University of the Witwatersrand and the Aga Khan University Institutional Scientific and Ethics Review Committee. Data preparation and model development are underway. Participatory workshops and DCEs are ongoing. Simulation-based validation and prototype refinement will follow.

**Conclusions:**

AfriCAT aims to develop the first adaptive mental health assessment calibrated using nationally representative African adolescent data. By integrating precision psychometrics with participatory co-design, the study seeks to enable more accurate triage and support scalable, measurement-based adolescent mental health care in resource-constrained settings.

## Introduction

### Background

Mental health conditions are among the leading causes of disability among adolescents globally [1]. Depression and anxiety disorders contribute substantially to the global burden of disease in young people and are associated with impaired functioning, reduced educational attainment, and increased risk of suicide [1,2]. In many low- and middle-income countries (LMICs), including those in sub-Saharan Africa, the majority of adolescents with mental health conditions do not receive appropriate care due to shortages of specialist providers, limited mental health literacy, stigma, and weak integration of mental health services within primary care systems [3].

To address these challenges, there has been growing interest in task-shared and stepped-care models of mental health service delivery, alongside the use of digital mental health interventions [4]. Task-sharing enables nonspecialist providers, including primary health care workers and trained lay counselors, to deliver evidence-based psychological interventions while reserving specialist services for more severe or complex cases [5]. Digital tools, including mobile- and web-based platforms, have also been explored as scalable approaches to delivering low-intensity mental health support [6-8]. Within stepped-care systems, these approaches can complement one another by enabling adolescents to access different levels of care based on symptom severity and clinical need [9]. However, effective implementation of such models requires efficient and scalable assessment tools to support identification, treatment planning, and monitoring of treatment response.

Measurement-based mental health care (MBMH) refers to the systematic use of standardized assessments to inform clinical decision-making across the care pathway [10,11]. Within MBMH frameworks, symptom measures are used not only to identify individuals who may require care but also to guide treatment selection and monitor symptom change over time [10-12]. Digital technologies increasingly enable such assessments to be embedded within scalable care platforms, facilitating self-monitoring and supporting clinical decision-making during consultations [9]. Although evidence from high-income settings suggests that measurement-based approaches can improve clinical outcomes, implementation in LMICs remains limited [12,13]. One reason is that commonly used assessment tools may be inefficient or poorly suited to repeated measurement in resource-constrained health systems [14].

Most widely used screening instruments, including the Patient Health Questionnaire-9 and Generalized Anxiety Disorder-7 scales, are static questionnaires that administer the same set of items to all respondents regardless of symptom severity [15,16]. Although brief and easy to administer, these instruments offer limited measurement precision across the full spectrum of symptom severity and can become burdensome when used repeatedly for treatment monitoring [17,18]. These limitations are compounded by the high degree of comorbidity between depression, anxiety, and other mental health conditions, requiring administration of multiple disorder-specific symptom scales [19]. Many existing digital mental health assessment tools similarly replicate static paper-based questionnaires, administering identical item sets irrespective of individual symptom profiles, meaning that digital delivery alone does not necessarily improve the efficiency or utility of assessments [20].

Computerized adaptive testing (CAT) offers an alternative approach to mental health assessment [20-22]. CAT uses statistical models, typically derived from item response theory, to dynamically select questions that provide the greatest informational value for each respondent [21]. As a result, adaptive tests can achieve measurement precision comparable to full-length instruments while administering substantially fewer items [21,23]. Adaptive testing approaches have been successfully applied to the assessment of depression, anxiety, and other psychiatric symptoms, demonstrating improved efficiency without compromising diagnostic accuracy [24,25]. Further, by drawing from large item banks spanning multiple symptom domains, they can estimate underlying dimensional constructs that cut across diagnostic categories and may therefore provide a more efficient means of assessing multiple mental health conditions simultaneously [21]. The adaptive item selection of CAT-based measurement also helps reduce familiarity-related responder bias, as individuals are less likely to encounter the same fixed set of items during repeated assessments.

Despite these advances, adaptive mental health assessments have largely been developed using data from high-income countries, with limited representation of adolescents from African settings. Moreover, adaptive assessments have rarely been explicitly conceptualized as tools to support measurement-based mental health care within resource-constrained health systems. Evidence is also limited regarding the feasibility, acceptability, and appropriateness of adaptive mental health assessments among adolescents in African contexts or how such tools might be integrated into existing stepped-care service models.

### Conceptual Framework

The AfriCAT study was designed to address these gaps by developing a computerized adaptive assessment for depression and anxiety among adolescents using nationally representative data from Kenya. Importantly, AfriCAT is conceptualized not as a screening tool alone but as a scalable adaptive assessment engine embedded within a stepped-care system to support measurement-based mental health care.

**Figure 1.**
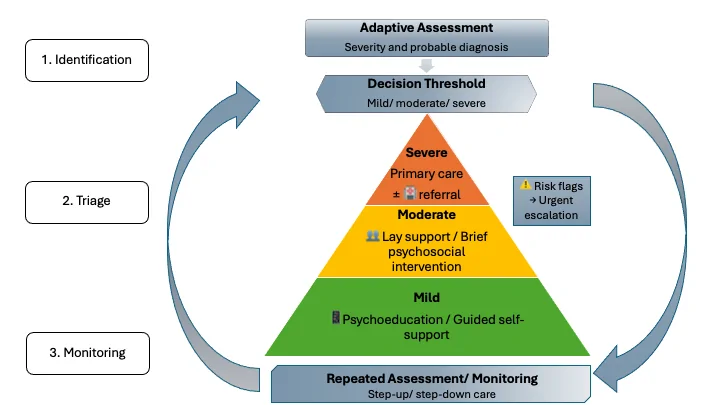
Adaptive assessment within a stepped-care, measurement-based mental health care framework.

Within this framework (Figure 1), adaptive assessment contributes to 3 core functions across the care pathway. First, it supports identification by estimating the probability of depressive and anxiety disorders and generating dimensional severity scores. Second, these outputs inform triage, enabling stratification of individuals into levels of care based on symptom severity and risk, ranging from low-intensity, high-volume interventions to more intensive, clinician-supported services. Third, repeated adaptive assessments enable measurement-based care, allowing providers to monitor symptom trajectories and adjust treatment through step-up or step-down transitions over time.

Importantly, the framework incorporates risk-responsive pathways, whereby elevated risk (eg, suicidality) triggers immediate escalation to higher levels of care. In this way, adaptive testing functions as a continuous decision-support mechanism, informing both initial triage and ongoing management within resource-constrained, task-shared systems.

This figure illustrates how adaptive assessment supports measurement-based care through identification, triage, and ongoing monitoring within a stepped-care system. Individuals are stratified into levels of care based on symptom severity and risk, with low-intensity, high-volume interventions at the base and higher-intensity, clinician-supported care at the apex. Repeated assessment enables dynamic step-up and step-down transitions over time. Risk flags (eg, suicidality) trigger immediate escalation pathways.

### Study Aim and Objectives

The overall aim of the AfriCAT study is to develop and internally validate a computerized adaptive assessment for adolescent depression and anxiety designed to support measurement-based mental health care in African contexts. Specific objectives are as follows:

1. to develop and calibrate adaptive assessment models for depression and anxiety using nationally representative adolescent mental health survey data.
2. to evaluate the precision, efficiency, and diagnostic classification performance of the adaptive assessment through simulation-based internal validation.
3. to compare adaptive algorithms derived from multidimensional item response theory (MIRT) and modular clinical decision support network (MODN) architectures.
4. to incorporate adolescent perspectives through participatory workshops and discrete choice experiments (DCEs) to inform tool design and implementation.
5. to assess stakeholder perspectives regarding the feasibility and potential integration of adaptive assessment into existing mental health systems.

## Methods

### Study Design

AfriCAT is designed as a multicomponent mixed methods study that integrates psychometric modeling, adaptive algorithm development, and participatory co-design.

The first component involves secondary analysis of nationally representative survey data to calibrate and simulate adaptive assessment models. In parallel, 2 complementary adaptive approaches will be developed: a MIRT-based framework grounded in latent trait estimation [26], and a MODN architecture designed to support sequential, consultation-aligned diagnostic updating [27].

Building on these adaptive engines, digital prototypes will be developed for both adolescent self-administration and provider use within primary care settings using task-sharing. The development process will be informed by participatory workshops with adolescents who have lived experience of depression and anxiety, as well as DCEs designed to quantify user preferences regarding key design and implementation features. In addition, semistructured interviews with mental health providers, primary health care workers, and policymakers will explore feasibility, workflow integration, and system-level considerations for scale-up. Reporting will align with established guidance on predictive modeling, participatory engagement, and mixed methods integration [28-30].

**Figure 2.**
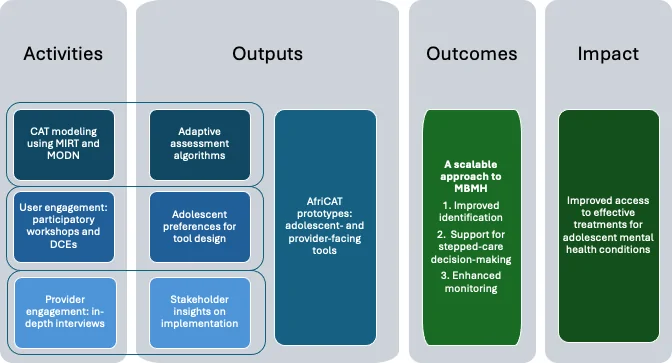
Logic model for the AfriCAT study. CAT: computerized adaptive testing; DCE: discrete choice experiment; MBMH: measurement-based mental health care; MIRT: multidimensional item response theory; MODN: modular clinical decision support network.

This study does not constitute a clinical trial. The present protocol focuses on tool development and internal validation within existing data. External validation in independent samples, including prospective clinical validation studies in Kenya and South Africa, will be undertaken in subsequent research before broader implementation is considered (Figure 2).

AfriCAT consists of three workstreams: (1) CAT modeling using MIRT and MODN; (2) engagement with adolescents with lived experience through participatory workshops and DCEs; and (3) engagement with mental health providers and policymakers through in-depth interviews. Outputs will be integrated into the AfriCAT prototypes. Further research will address the intended outcomes and impact.

### Lived Experience

Incorporating adolescents’ lived experiences of depression and anxiety is essential to ensure the tool reflects how young people experience and articulate their symptoms [31]. In this study, lived experience is defined as personally identifying as having experienced depression and/or anxiety; a formal clinical diagnosis is not required. Lived experience is embedded within AfriCAT at multiple levels. Youth Liaisons in Kenya and South Africa with lived experience are incorporated into the study team as coresearchers, contributing to study design, refinement of research materials, data collection, interpretation of findings, and dissemination. In addition, adolescents with lived experience are engaged in a series of participatory workshops and DCEs in Kenya and South Africa. Together, these approaches ensure that youth perspectives shape tool development, strengthening the acceptability and contextual relevance of the adaptive assessment.

### Study Settings

Participatory workshops and DCEs will be conducted in Nairobi, Kenya, and in 2 settings in South Africa: Agincourt (Mpumalanga Province) and Johannesburg. These sites reflect diverse urban and rural contexts within sub-Saharan Africa where adolescent mental health needs are substantial and service capacity remains constrained [32-34]. Mental health literacy among adolescents and caregivers varies considerably, and stigma continues to influence help-seeking behavior across both countries [35,36]. At the same time, both Kenya and South Africa have experienced rapid expansion in mobile phone access and digital connectivity among young people, creating opportunities for scalable digital mental health interventions [37,38]. Kenya has been recognized as a regional leader in mobile health innovation, while South Africa has articulated national digital health strategies that support integration of digital tools within primary care systems [39,40]. These contextual factors make Nairobi, Agincourt, and Johannesburg particularly relevant settings for examining the acceptability, feasibility, and implementation potential of a computerized adaptive assessment designed to advance measurement-based adolescent mental health care in resource-constrained systems.

### Data Source

The AfriCAT study will use data from the Kenya National Adolescent Mental Health Survey (K-NAMHS), a nationally representative household survey of adolescent girls (53.1%) and boys (49.9%) aged 10 to 17 years and their primary caregivers [34]. The large, population-based sample, standardized diagnostic instrument, and breadth of symptom items in K-NAMHS provide a rich item pool suitable for CAT development. The survey used a multistage stratified cluster sampling design to recruit adolescent–primary caregiver pairs from households across Kenya [34]. Mental health conditions were assessed using the *Diagnostic Interview Schedule for Children, version 5* (*DISC-5*), administered by trained lay interviewers, with modules covering major depressive disorder (MDD), generalized anxiety disorder (GAD), social phobia (SoPh), posttraumatic stress disorder (PTSD), attention-deficit/hyperactivity disorder, and conduct disorder [41]. *DISC-5* scoring algorithms are used to generate diagnoses aligned with the *Diagnostic and Statistical Manual of Mental Disorders, Fifth Edition* (*DSM-5*) [41]. Additional modules captured demographic characteristics, risk and protective factors, and service use. The final sample size of K-NAMHS was 5155 primary caregiver-adolescent pairs, with a response rate of 97.9%. Although K-NAMHS provides one of the few nationally representative adolescent mental health datasets currently available from sub-Saharan Africa, adaptive models developed from these data will require external validation in other populations before broader generalization across African settings can be assumed.

### Item Bank Development

The AfriCAT item bank will be constructed from symptom-level items within the *DISC-5* modules assessing MDD, GAD, SoPh, and PTSD. These conditions represent the most common internalizing mental health conditions in adolescence and are characterized by substantial symptom overlap and comorbidity, making them well suited to dimensional measurement approaches [19]. These modules were also administered directly to adolescents (as opposed to caregivers), reducing complexity for purposes of developing an adaptive assessment [42]. Externalizing modules (eg, attention-deficit/hyperactivity disorder and conduct disorder) were excluded to maintain a coherent internalizing measurement framework.

### MIRT-Based CAT Development

To capture the latent structure of symptoms appropriately, a bifactor multidimensional framework is outright adopted for model calibration. Within this definitive framework, all items load directly onto a single general internalizing factor while simultaneously contributing to independent, disorder-specific factors corresponding to MDD, GAD, SoPh, and PTSD. Model fit for this bifactor configuration will be rigorously assessed by evaluating the strength and significance of item factor loadings, examining global fit statistics, specifically the limited-information M2 statistic alongside absolute and relative indices such as the root mean square error of approximation and the comparative fit index, and visually inspecting observed versus expected item response plots to identify localized areas of measurement.

*DISC-5* symptom items in the K-NAMHS dataset are recorded as binary (yes/no) responses, indicating the presence or absence of specific symptoms. Item parameters will therefore be estimated using dichotomous item response theory models, allowing estimation of item discrimination and severity parameters for each symptom indicator. The baseline MIRT bifactor calibration will be executed via a stochastic expectation-maximization algorithm. To ensure population representativeness, normalized complex survey weights will be integrated directly into the estimation process to scale individual contributions to the marginal likelihood. Missing data handling strategies will differ by analytical phase. For the core MIRT calibration, missingness will be accommodated via full-information maximum likelihood, which leverages all available structural information without data loss. However, because post hoc adaptive testing simulations within the MIRT-CAT framework require a complete matrix to execute through high-missingness skip patterns, model-based plausible value imputation will be utilized strictly to construct the complete simulation response bank.

Adaptive simulations will iteratively select items that maximize information at the current trait estimate and allow us to determine the extent to which the CAT reproduces information in the full item bank. Item administration within the CAT engine will cease when the SE of the general factor theta estimate falls below a specified precision threshold, which is optimized to maximize the posttest correlation coefficient with full-bank estimates. Alternatively, administration will stop when a maximum question cap is reached to minimize respondent burden, allowing us to balance measurement precision with assessment length.

Model performance and operational efficiency will be evaluated through post hoc adaptive simulations executed across the entire calibrated dataset, adhering strictly to established psychometric validation standards for multidimensional screening tools.

### MODN

In parallel with the MIRT-based development, AfriCAT will also be implemented using a MODN architecture [27]. MODN is a modular neural network framework in which each symptom question functions as an independent module that updates diagnostic probability estimates sequentially as new information is obtained [27]. Rather than estimating a single latent trait, this approach models the evolving probability of specific diagnostic states, recalibrating predictions dynamically with each response. Unlike traditional latent trait models, MODN more closely mirrors the logic of a clinical consultation, in which information is gathered iteratively and diagnostic impressions are updated in real time.

A deep learning encoder module will be created for each question in the dataset. A deep learning decoder module will be created for each outcome. Missing data are accommodated by the modular architecture and are simply ignored.

Five-fold cross-validation will be performed on the training set to optimize the hyperparameters of the models. The final models will then be trained on the full training set with the optimal hyperparameters. All analyses will be performed using the trained models on the test set. Overfitting will be controlled by early stopping the training after increases in the validation loss and sanity-checking the loss curves.

To remedy the low prevalence of internalizing disorders, we will also train models that overweight positive diagnosis cases. As the survey weights do not substantially alter the prevalence of the internalizing disorders, the models trained using them are not expected to differ significantly and would be less representative of individual adolescents. We will therefore not use the survey weights.

The model achieving the best prediction performance using all the questions on the test set will then be selected. Different combinations of next-question-selection methods and stopping rules will be compared based on their predictive performance and efficiency. They will be compared against baselines using entropy-based methods as next-question-selection and the *DISC-5* diagnostic criteria satisfaction as the stopping rule.

### Prototype Development

Insights from both the MIRT- and MODN-based approaches will inform final prototype design, including the possibility of hybrid architectures that combine strengths of probabilistic prediction and latent severity estimation. Two complementary user interfaces will be developed to reflect the different contexts in which AfriCAT may be used within measurement-based mental health care systems.

The first is an adolescent-facing interface designed for self-administration, for example, via smartphone or tablet in school, community, or clinic settings. This version will deliver symptom questions adaptively, presenting items sequentially based on prior responses to minimize burden while maintaining precision. Upon completion, the tool will provide structured feedback tailored to symptom severity alongside guidance regarding appropriate referral or support options.

The second interface is a provider-facing version intended for use by primary health care workers, mental health professionals, or trained lay providers within task-shared systems of care. In this format, the adaptive assessment will be designed for integration into the clinical encounter, supporting real-time evaluation of symptom severity. The tool will be designed to generate dimensional severity estimates and probabilistic diagnostic outputs that can inform clinical decision-making. The prototypes developed during this study are intended for research and feasibility testing only and will not be used to make independent clinical decisions or provide automated crisis management recommendations.

### Participatory Workshops

Participatory workshops will ensure that AfriCAT is grounded in the perspectives and priorities of young people and that the resulting prototypes are acceptable, comprehensible, and contextually appropriate. A total of four participatory workshops will be conducted in Nairobi, Kenya, and Agincourt, South Africa (two in each country). During these workshops, approximately 50 young people aged 10 to 24 years will engage in focus group discussions, guided by semistructured interview guides (Multimedia Appendix 1).

The first workshops will explore the acceptability of adaptive assessment approaches, including perceptions of dynamically changing questions and the tone and framing of feedback provided by the tool. Adolescents will also be invited to reflect on privacy expectations, data-sharing concerns, and preferences regarding who should have access to their assessment results. In addition, discussions will examine preferred implementation contexts, such as schools, clinics, universities, or home-based digital platforms. The final workshops will involve prototype testing, completion of validated feasibility and acceptability quantitative surveys (Multimedia Appendix 1), and a focus group discussion to further unpack adolescents’ perceptions of the AfriCAT tool and reflect on their experiences of involvement in the co-design process.

### DCE

To complement qualitative data, a DCE will be conducted to quantify adolescent preferences regarding key design and implementation features of AfriCAT. A total of 500 adolescents aged 12 to 24 years will be recruited from schools, communities, and universities across Kenya and South Africa. The DCE will focus on five attributes considered central to tool design: (1) where the test can be taken (access pathway), (2) how questions about personal information are handled (privacy of demographics), (3) who can see the results (results sharing), (4) how much and what information appears on the results page, and (5) the description of the tool on the landing page. Each attribute will be presented at 2 or 3 levels, described in simple language familiar to adolescents (Multimedia Appendix 1).

In total, 20 choice tasks will be created, each consisting of 2 alternative versions of the tool that differ in how the attributes are combined. Participants will be presented with a series of hypothetical scenarios in which alternative versions of AfriCAT differ across these attributes. For each pair of scenarios, adolescents will indicate their preferred option. This approach allows estimation of the relative importance placed on different features and the trade-offs adolescents are willing to make between them. To minimize burden and reduce fatigue, these tasks will be divided into 2 blocks of 10 tasks each. Participants will be randomly assigned to group A or group B, with each group completing one block (10 tasks). Within each task, participants will be asked to select which of the 2 versions they would prefer to use. Randomization ensures that participants are evenly distributed across the 2 blocks, while still allowing researchers to collect sufficient data for robust analysis. The DCE design will follow best practice principles of experimental design, using a D-efficient approach to maximize the information gained from each participant’s choices. Dominance checks and attention filters will be included to monitor data quality.

Choice modeling techniques, such as conditional or mixed logit models, will be used to estimate preference weights and to examine whether preferences vary by age group, gender, or country. Findings from the DCE will inform refinement of the prototypes and support implementation strategies that align with adolescent priorities.

### Stakeholder Interviews

To complement adolescent perspectives, approximately 20 to 24 semistructured interviews will be conducted with mental health providers, primary health care workers, and policymakers in Kenya and South Africa following a semistructured interview guide (Multimedia Appendix 1). These interviews will explore how AfriCAT could be integrated into existing health and education systems, with particular attention to feasibility within resource-constrained environments. Discussions will examine alignment with stepped-care protocols, potential roles within task-shared service models, evidence requirements for broader adoption or policy endorsement, and perceived resource implications, including workforce and training considerations. Insights generated through this process will inform the development of an implementation strategy and ensure that AfriCAT is responsive not only to adolescent preferences but also to system-level realities.

### Ethical Considerations

Ethical approval for the AfriCAT study has been obtained from the Human Research Ethics Committee (Medical) at the University of the Witwatersrand (MED 25-06-1023) and the Aga Khan University Institutional Review Board (2025/ISERC-147). The study will be conducted in accordance with the Declaration of Helsinki and relevant data protection legislation in South Africa and Kenya. The secondary data analysis component uses deidentified individual-level data from K-NAMHS, and no attempts will be made to reidentify participants. Data will be stored securely on institutional servers, with access restricted to authorized study personnel and managed in compliance with applicable data protection policies.

The participatory components involve adolescents who self-identify as having lived experience of depression and/or anxiety and are therefore considered a vulnerable group. Adolescents aged 13 to 17 years will provide written assent and parental consent, while participants aged 18 years and older will provide written informed consent. Information sheets will emphasize the voluntary nature of participation, the right to withdraw at any time, and the absence of consequences for nonparticipation. A study-specific distress protocol (Multimedia Appendix 1) has been developed to guide the identification and management of participant distress.

Participants who disclose suicidal ideation, significant psychological distress, risk of harm to self or others, abuse, or other safeguarding concerns during study activities will be managed according to the study-specific distress protocol. Facilitators conducting workshops, interviews, and prototype testing will be trained in recognizing and responding to participant distress. Where immediate risk is identified, participants will be referred to appropriate local mental health, social, or emergency services in accordance with site-specific referral pathways. For participants younger than 18 years, parental or guardian involvement will occur where required for participant safety and in accordance with local ethical requirements and applicable laws. Workshops and interviews will be facilitated by trained researchers, including team members with clinical experience in adolescent mental health.

Confidentiality will be strictly maintained through the use of unique study identification numbers and secure storage of identifiable information separate from research data. Audio recordings will be deidentified during transcription and deleted once accuracy is verified. Although confidentiality within focus group discussions cannot be fully guaranteed, participants will be informed of these limitations and encouraged to respect the privacy of others. Participants will be informed that confidentiality may be breached where there is concern regarding imminent risk of serious harm to themselves or others, or where disclosure is required by law or ethical obligations relating to child protection. The AfriCAT prototypes will not be deployed clinically during this phase of research; any future implementation or large-scale use will undergo additional ethical review.

### Outcomes

#### Primary Outcomes

Because MIRT and MODN estimate fundamentally different quantities, namely severity and probability of diagnosis, they cannot be meaningfully compared head-to-head. Instead, we will review the relative strengths and limitations of the models in supporting measurement-based mental health care. Primary metrics of evaluation will include (1) precision of severity estimation across internalizing symptom dimensions; (2) efficiency, operationalized as the median number of items required to achieve stable estimates or certain predictive performance; and (3) diagnostic classification performance relative to *DSM-5* diagnoses for MDD, GAD, SoPh, and PTSD. Diagnostic classification performance will be evaluated using established metrics such as sensitivity, specificity, and area under the receiver operating characteristic curve. To assess fairness across age and gender, each outcome will be computed for each subgroup and compared.

#### Secondary Outcomes

Secondary outcomes include evaluation of the adaptive assessment’s ability to estimate a general internalizing dimension alongside disorder-specific symptom domains, as well as assessment of model stability under internal validation procedures. For the MODN architecture, the calibration of probabilistic outputs will be assessed using calibration curves and the expected calibration error metric. The interpretability of sequential prediction updates will be represented as heatmaps and will be qualitatively assessed for potential integration into routine provider workflow.

For the DCE, outcomes will include estimation of relative attribute importance and trade-offs between implementation features. Preference heterogeneity by country and age group will also be explored. Quantitative surveys within the second workshops with adolescents will explore feasibility, acceptability, and appropriateness of the AfriCAT tool using validated scales [43]. For the qualitative components, outcomes will include identification of themes related to acceptability, feasibility, appropriateness, and alignment with stepped-care frameworks.

### Analysis

Analysis and modeling will be conducted using R and Python [44,45]. The K-NAMHS dataset will be split into a training set containing 80% of the data and a held-out test set containing 20% of the dataset. The split will be stratified on the outcomes so the proportions of records with certain outcomes are the same in the training and test sets.

Survey data will be analyzed using Stata 19 [46]. Descriptive statistics will be used to summarize participant characteristics and survey responses, with categorical variables reported as frequencies and percentages and continuous variables summarized using means and SDs or medians and IQRs, as appropriate. DCE outcomes will be assessed using conditional or mixed logit models within a random utility framework.

Qualitative data from workshops and interviews will be audio-recorded, transcribed verbatim, and analyzed using framework analysis, a structured form of thematic analysis commonly used in applied health and policy research [47,48]. This approach enables systematic comparison of perspectives across participant groups while accommodating both deductive and inductive coding. An initial analytic framework was developed based on study objectives and key domains relevant to tool design and implementation (Multimedia Appendix 1) and will be iteratively refined through close engagement with the data. Interpretation will focus on identifying patterns and insights relevant to the acceptability, usability, and potential integration of AfriCAT within existing adolescent mental health systems.

Results will be interpreted in relation to the study’s overarching objective of supporting scalable measurement-based adolescent mental health care. The study adopts a pragmatic epistemological orientation, prioritizing knowledge that is both methodologically rigorous and applicable within real-world health systems. Quantitative psychometric modeling will evaluate the measurement precision, efficiency, and diagnostic performance of the adaptive assessment, while qualitative and preference-based methods will examine acceptability, usability, and implementation considerations. Reflexivity will be incorporated throughout the qualitative components, with researchers reflecting on how their roles, disciplinary perspectives, and relationships with participants may influence data generation and interpretation. Findings will be integrated using a convergent mixed methods approach [49], combining modeling results with insights from participatory workshops, DCEs, and stakeholder interviews to assess both statistical performance and real-world feasibility of the AfriCAT prototypes.

This protocol is based on a competitively peer-reviewed and funded proposal, with refinements informed by reviewer feedback; the original reviewer reports are provided in Peer Review Report 1.

## Results

Data preparation and the psychometric modeling component are complete, and calibration of the adaptive models is underway. Development of the MIRT- and MODN-based architectures is in progress. Participatory workshops with adolescents and DCE data collection are ongoing in Kenya and South Africa. Simulation-based internal validation of the adaptive algorithms and iterative refinement of the adolescent-facing and provider-facing prototypes will follow completion of model calibration and analysis of participatory findings.

## Discussion

### Expected Outcomes

The primary expected outcome of AfriCAT is the development and internal validation of a computerized adaptive assessment for adolescent depression and anxiety calibrated using nationally representative Kenyan adolescent mental health data. Subsequent research will evaluate the transportability and performance of the adaptive assessment in other African settings. The study will generate new knowledge regarding the dimensional structure of internalizing symptoms in Kenyan adolescents, the feasibility of adaptive assessment within this context, and the comparative performance of MIRT-based and modular neural architectures for mental health evaluation. By integrating psychometric modeling with participatory co-design and quantified preference elicitation, the study will also contribute methodologically to the emerging field of digital mental health in LMICs.

Beyond advancing psychometric science, AfriCAT is intended to serve as foundational infrastructure for measurement-based adolescent mental health care. The adaptive engine is designed to support identification of clinically significant symptoms, stratification within stepped-care models, and longitudinal monitoring of treatment response. In resource-constrained systems, where specialist capacity is limited and task-shared models are required, efficient and precise assessment tools are essential for optimizing allocation of care. By reducing assessment burden while maintaining measurement precision, AfriCAT has the potential to support scalable screening and monitoring without overwhelming services.

### Comparison With Prior Work

CAT for mood and anxiety assessment has a substantial evidence base in high-income settings, demonstrating that precise severity estimation and accurate case identification can be achieved with markedly reduced respondent burden [25]. Early foundational work by Gibbons et al [50] developed adaptive depression assessment (CAT-Depression), showing strong concordance with full item-bank scores and clinically meaningful discrimination while substantially shortening administration time. Related work extended this approach to anxiety via CAT-Anxiety, supporting the feasibility of adaptive methods for rapid and accurate assessment of GAD and comorbid presentations [51]. In parallel, the Patient-Reported Outcomes Measurement Information System pediatric item banks for anxiety and depressive symptoms established an item response theory–calibrated measurement infrastructure that supports short forms and simulated CAT administration for youth populations, further underscoring the viability of adaptive assessment for adolescent mental health [52]. More recently, multidimensional CAT approaches have been developed to assess a broader range of youth psychopathology dimensions, highlighting the potential of adaptive tools to support scalable and clinically informative measurement in children and adolescents [53].

Despite these advances, the adaptive mental health assessment literature remains largely dominated by data and calibration samples from high-income countries and has seldom been explicitly positioned as enabling infrastructure for measurement-based mental health care within resource-constrained health systems. AfriCAT addresses this gap by developing an adaptive assessment calibrated using nationally representative African adolescent data and by pairing psychometric development with participatory design, preference elicitation, and implementation-focused stakeholder engagement. In addition, AfriCAT extends prior work by exploring not only latent trait–based adaptive testing (MIRT) but also a MODN, which has been proposed as an updatable and interpretable framework for sequential decision-making in evolving clinical environments and may be particularly well suited to provider-facing deployment in stepped-care and task-shared settings.

### Strengths

The study has several strengths. It leverages nationally representative data, enhancing generalizability and public health relevance. It integrates complementary adaptive modeling approaches (MIRT and MODN), allowing comparison of latent trait–based and modular predictive architectures. Lived experience is embedded both through participatory engagement and through Youth Liaisons incorporated as coresearchers, strengthening contextual validity. The inclusion of DCEs provides quantitative evidence on user preferences, supporting implementation readiness. Finally, the study explicitly situates adaptive assessment within a measurement-based care framework, linking methodological innovation to health system application.

### Limitations

The study also has limitations. Calibration and validation are based on secondary, cross-sectional survey data, which may not fully capture longitudinal symptom trajectories relevant to treatment monitoring. Further, the item pool is limited to items used in the *DISC-5* diagnostic interview. Although the dataset is nationally representative of Kenyan adolescents, the adaptive models are calibrated using data from a single country and therefore may not fully capture cultural, linguistic, epidemiological, and health system differences across African settings. Generalizability beyond Kenya cannot be assumed and will require external validation in independent populations. Planned future studies include prospective clinical validation in Kenya and South Africa, with potential extension to additional African countries through existing collaborative partnerships.

Simulation-based evaluation cannot fully substitute for prospective real-world testing, particularly in relation to feasibility, usability, safety, and clinical impact. Future implementation studies will need to evaluate safety procedures, referral workflows, management of suicide risk, data governance requirements, and integration with local safeguarding systems across different deployment contexts. In addition, adaptive models derived from structured diagnostic interviews may require refinement, particularly when implemented in self-administered digital formats. These limitations will be addressed in subsequent phases of research involving field validation and implementation studies.

### Dissemination

The results of this study will be disseminated through peer-reviewed publications, conference presentations, plain language summaries, and stakeholder engagement activities. Findings will inform future implementation and validation studies, including prospective field testing within primary care and school-based platforms. Engagement with policymakers and health system stakeholders during the study will facilitate translation of findings into programmatic decision-making, particularly in relation to stepped-care frameworks and digital health strategies. Ultimately, AfriCAT is intended to contribute to strengthening adolescent mental health systems in African settings by enabling more systematic, data-informed, and equitable approaches to identification and care.

In line with principles of open science and reproducibility, statistical code used for psychometric modeling, adaptive simulation, and preference analysis will be made publicly available through an open-access repository (eg, GitHub and archived via Zenodo) upon publication of primary results. Deidentified derived datasets generated during the study will also be made available through a public data repository. Because the K-NAMHS dataset is governed by data-sharing agreements and institutional oversight, access to the original individual-level survey data will be provided through the data custodians in accordance with established application procedures.

### Conclusion

AfriCAT seeks to advance measurement-based adolescent mental health care in Africa through development of a computerized adaptive assessment engine. By embedding psychometric innovation within participatory and implementation-oriented methods, the study aims to contribute to scalable, efficient, and contextually grounded mental health assessment.

## Supplementary material

10.2196/98369 Multimedia Appendix 1 Study materials and analytic framework supporting the AfriCAT protocol.

10.2196/98369 Peer Review Report 1 Peer review report by the Mental Health Data Prize – Africa (MHDP-A), Africa Population and Health Research Center (APHRC).

## Abbreviations

CAT: computerized adaptive testing
DCE: discrete choice experiment
*DISC-5*: *Diagnostic Interview Schedule for Children, version 5*
*DSM-5*: *Diagnostic and Statistical Manual of Mental Disorders, Fifth Edition*
GAD: generalized anxiety disorder
IRT: Item Response Theory
K-NAMHS: Kenya National Adolescent Mental Health Survey
LMIC: low- and middle-income country
MBMH: Measurement Based Mental Healthcare
MDD: major depressive disorder
MIRT: multidimensional item response theory
MODN: modular clinical decision support network
PTSD: posttraumatic stress disorder
SoPh: social phobia
