# Supplementary material for: Measurement-Based Mental Health Care for Adolescents in Africa: Protocol for a Computerized Adaptive Test
Source: JMIR Res Protoc. 2026 Sep 8;15:e98369. doi: 10.2196/98369 (PMC13552835; doi:10.2196/98369)
Supplement: Supplementary file 1 [file resprot-v15-e98369-s001.docx]

Multimedia Appendix 1

# Study materials and analytic framework supporting the AfriCAT protocol

Table of Contents

[Appendix 1: DCE Attributes and levels	2](#_Toc227157453)

[Appendix 2. Focus Group Discussion Guide	4](#_Toc227157454)

[Appendix 3. Analysis Framework	8](#_Toc227157462)

[Appendix 4: Acceptability, Appropriateness and Feasibility Scales	10](#_Toc227157463)

[Appendix 5: Stakeholder Interview Guide	11](#_Toc227157464)

[Appendix 6. Distress Protocol	16](#_Toc227157465)

## Appendix 1: DCE Attributes and levels

| ***Attribute*** | ***Levels*** | ***Levels (verbatim)*** |
| --- | --- | --- |
| ***Where you take the test (Access pathway)*** | 3 | 1. App X is a tool you **can use anywhere,** such as at home, at school, or in a clinic. 2. App X is a tool that you **use only if a counsellor or health worker refers you,** and you do it **during your session with them.** 3. App X is a tool that you **use only if a counsellor or health worker refers you,** and you **can use anywhere,** such as at home, at school, or in a clinic. |
| ***Privacy about a few questions about you (Handling of demographics)*** | 3 | 1. It **is optional not** to answer questions about your age, gender or where you live. 2. It **is optional** to answer questions about your age, gender or where you live. 3. It **does not ask you** questions about your age, gender or where you live. |
| ***Who sees your results (Results sharing)*** | 3 | 1. **Only you can see and download your results.** 2. **You can see and download your results**, which are **automatically sent to a health worker** or counsellor. 3. **Only you can see and download your results**. You also have the option to share your results with a health worker or counsellor. |
| ***Landing-page description style*** | 3 | 1. On the **home page, App X is described as a brief assessment** of depression and anxiety that should **take 3-5 minutes to complete** 2. On the home page, **App is described as a brief assessment** of depression and anxiety **that uses AI** to ask the most relevant questions about your mental health, that **should take 3-5 minutes to complete** 3. On the **home page, App is described as a brief assessment** of depression and anxiety **that uses AI** to ask the most relevant questions about your mental health. |
| ***How much information on the results page*** | 2 | 1. The **results page tells you if you have symptoms** of depression or anxiety and how severe they are. The tool will **also link to resources** on how to manage your mental health and **advise you to repeat the assessment in a week or two.** 2. The **results page tells you if you have symptoms** of depression or anxiety and how severe they are **and whether it is recommended to contact a counsellor or** health worker. The tool will **also link to resources** on how to manage your mental health and **advise you to repeat the assessment in a week or two.** |

Table A1. Attributes, levels and tasks included in the Discrete Choice Experiment (DCE) for AfriCAT. The table outlines five key attributes: access pathway, handling of demographics, results sharing, results page detail, and landing-page description style and the number of levels and verbatim descriptions presented to participants.

**Design Parameters**

| ***Parameter*** | ***Value*** | ***Notes*** |
| --- | --- | --- |
| *Alternatives per task (J)* | **2** | Profile A vs Profile B |
| *Tasks per respondent (T)* | **10** | Assigned via Block A **or** Block B |

## Appendix 2. Focus Group Discussion Guide

### Session plan

| **Module** | | **Focus** |
| --- | --- | --- |
| 1. | Welcome and ground rules | Consent, confidentiality, comfort check |
| 2. | Warm-up: language and help seeking | How young people describe distress and seek help |
| 3. | First reactions to the CAT idea | Acceptability and understanding of purpose |
| 4. | Deep dive into user journey | Access, privacy, questions, results , sharing , safeguarding |
| 5. | Barriers and bottlenecks | Practical constraints and implementation risks |
| 6. | Prioritization and close | Must-haves, deal breakers, final message to designers |

### 1. Welcome and ground rules

**Objective:** Create a safe, respectful environment and explain the purpose of the discussion.

**Suggested wording:**

- Thank you for joining today’s workshop. We are designing a digital self-check tool for mental health. We want your opinions on what would feel helpful, safe and easy to use.
- There are no right or wrong answers. Please share your views freely.
- Let’s talk set some ground rules together for how we want to engage as a group (cover respect, confidentiality, dealing with distress) .

### 2. Warm up: Language and help-seeking

**Objective :** Understand local language/tone, typical help-seeking and barriers that may shape tool design.

#### Q1. When young people in your community talk about stress, sadness or anxiety, what words or phrases do they use?

Probes:

- What words describe mental health challenges?
- Are there different words used by young people vs adults?
- Are there words that feel stigmatizing or insulting vs. others which feel respectful?

#### Q2. When someone your age is struggling, what do they usually do first? Who do they talk to (if anyone)?

Probes:

- What makes it easier or harder to seek help (probe on things like privacy, money, stigma, fear of consequences)?
- Where do young people avoid going for help and why?
- If they cannot access help, what do they do instead?

### 2. First reactions to the CAT self-screening idea

**Objective:** Test initial acceptability, understanding, and perceived value of a digital self-screening CAT.

#### Q3. Imagine there is a phone or website tool that asks a small number of questions and gives you a result about your mental wellbeing. What is your first reaction?

Probes:

- What feels good about this idea? What feels worrying or uncomfortable?
- Who would this be for? When would someone use it?
- What would make you trust or mistrust it?

#### Q4. In your own words, what do you think this tool can and cannot do?

Probes:

- Some tests are like a quick check to see if something might be going on, and others are a full assessment by a professional. Where would you place this tool, and why?
- What would you expect to happen after getting a result?

### 3. User journey deep-dive

**Objective:** Gather concrete design preferences along the full journey: access, onboarding, privacy/data, question experience, results, sharing, and safeguarding.

**(3a) Access and onboarding**

#### Q5. How would you prefer to access the tool?

Probes:

- Would you prefer to access it anytime by yourself, only after referral (e.g., teacher/clinician), or during a session with someone? Why?
- Where would you realistically use it (home, school, clinic, cybercafé, shared space)?
- Would you prefer an app, a website link, WhatsApp/USSD style, or something else?

#### Q6. What would you want on the first screen/landing page to feel comfortable starting?

Probes:

- What information should be shown first (purpose, confidentiality, time to complete)?
- What design elements increase trust (logos, partners, ‘about’ page, evidence)?
- What would immediately make you close the page?

**(3b.) Privacy and data**

#### Q7. What privacy or confidentiality concerns would you have using this tool in real life?

Probes:

- Do young people share phones? Who might see the screen, notifications, or browsing history?
- Would you want features like a PIN, ‘quick exit’ button, or hiding results?
- What personal details (age, gender, location) feel okay to ask, and what is too much?

**(3c) Question experience (CAT)**

#### Q8. What kinds of questions would feel okay to answer on a phone, and what kinds would feel too personal or uncomfortable?

Probes:

- Should questions be short or long? Multiple-choice vs sliders vs emojis?
- What wording would feel judgemental vs supportive?
- How long is ‘too long’ for the whole assessment?

#### Q9. What would make someone stop halfway through the tool?

Probes:

- Data use, loading time, needing login, fear of being seen, unclear questions, upsetting content?
- What would help someone continue (progress bar, reassurance, ability to pause)?

**(3d) Results page and next steps**

#### Q10. After finishing, what would you want to see on the results page?

Probes:

- Would you prefer categories (e.g., mild/moderate/high) or a score? Why?
- What wording would feel supportive vs scary?
- What should the tool say to prevent people from thinking the result is a definite diagnosis?

#### Q11. What should the tool recommend you do next, based on the result?

Probes:

- Self-care tips? links to services? talking to someone? repeating the test later?
- What kinds of support are realistic in your area (school counsellor, clinic, helplines, peer support)?
- What would feel helpful immediately after seeing a concerning result?

**(3e) Sharing results**

#### Q12. Who (if anyone) should be able to see or receive the results?

Probes:

- Only me; optional sharing; automatically shared with a counsellor/clinician; shared with a parent/guardian?
- What sharing options feel safe and why?
- Would you want to download, screenshot, or send results?

**(3f) Safeguarding scenario**

#### Q13. If the tool detects someone may be at risk of harming themselves, what should happen next?

Probes:

- Should it show urgent help options (hotline/clinic), encourage contacting a trusted person, or connect to a live chat?
- When, if ever, is it acceptable for the tool to alert someone else automatically?
- What message would feel supportive in that moment?

### 4. Barriers, bottlenecks and ‘what could go wrong?’ (8–10 min)

**Objective:** Identify practical constraints, risks and implementation barriers for real-world uptake and sustained use.

#### Q14. What would stop young people from using this tool, even if it exists?

Probes:

- Phone access, data costs, low bandwidth, literacy/language, disability access?
- Fear of parents/teachers finding out?
- Not trusting the tool or fearing consequences?

#### Q15. What needs to be in place for this tool to work well in the real world?

Probes:

- If many young people screen ‘high’, who supports them?
- Which services or people should be connected to the tool, if any?
- What partnerships (schools, clinics, youth groups) would help adoption?

### 5. Prioritization & close

**Objective:** Get clear priorities for early product decisions and end on a positive, participant-centred note.

#### Q16. From everything we discussed, what are your top 3 MUST-HAVES for this tool?

Probes:

- If you could only keep three things, what would they be?

#### Q17. What are your top 3 DEAL-BREAKERS (things that would make you refuse to use it)?

Probes:

- What would make you close it immediately or never return?

#### Q18. If you could send one message to the designers/developers, what would it be?

## Appendix 3. Analysis Framework

Use this framework as a starting point to synthesise findings after each participatory workshop. Fill one row per domain with 2–3 key points and 1 illustrative quote. Then add ‘Design implications’ and ‘Implementation/bottleneck implications’ to inform prototype development

| **Domain** | **Key points (what we heard)** | **Illustrative quote** | **Design implications (MVP decisions)** | **Implementation/bottleneck implications** | **Priority** |
| --- | --- | --- | --- | --- | --- |
| Value proposition & use cases |  |  |  |  |  |
| Trust & legitimacy cues |  |  |  |  |  |
| Language & tone (preferred terms) |  |  |  |  |  |
| Access & onboarding (how/where used) |  |  |  |  |  |
| Privacy realities (shared phones, stealth features) |  |  |  |  |  |
| Data to collect (what is acceptable) |  |  |  |  |  |
| Assessment burden (time/items) & drop-off triggers |  |  |  |  |  |
| Question formats & wording do’s/don’ts |  |  |  |  |  |
| Results format (score vs categories) & comprehension risks |  |  |  |  |  |
| Next steps & support preferences |  |  |  |  |  |
| Sharing results preferences & red lines |  |  |  |  |  |
| Safeguarding expectations (risk scenarios) |  |  |  |  |  |
| Equity/accessibility (data, literacy, disability, language) |  |  |  |  |  |
| Wider context (stigma, adult reactions, school/clinic fit) |  |  |  |  |  |

## Appendix 4: Acceptability, Appropriateness and Feasibility Scales

| **Acceptability of Intervention Measure (AIM)** | | | | | | |
| --- | --- | --- | --- | --- | --- | --- |
| **1.** | **(The AFRICAT tool) meets my approval.** | Strong Disagree (1) | Disagree (2) | Neither Agree, or Disagree (3) | Agree  (4) | Strongly Agree  (5) |
| 2. | **(The AFRICAT tool) is appealing to me.** | Strong Disagree (1) | Disagree (2) | Neither Agree, or Disagree (3) | Agree  (4) | Strongly Agree  (5) |
| 3. | **I like (The AFRICAT tool).** | Strong Disagree (1) | Disagree (2) | Neither Agree, or Disagree (3) | Agree  (4) | Strongly Agree  (5) |
| 4. | **I welcome (the AFRICAT tool).** | Strong Disagree (1) | Disagree (2) | Neither Agree, or Disagree (3) | Agree  (4) | Strongly Agree  (5) |

| ***Intervention Appropriateness Measure (IAM)*** | | | | | | |
| --- | --- | --- | --- | --- | --- | --- |
| **1.** | **(The AFRICAT tool) seems fitting.** | Strong Disagree (1) | Disagree (2) | Neither Agree, or Disagree (3) | Agree  (4) | Strongly Agree  (5) |
| 2. | **(The AFRICAT tool) seems suitable.** | Strong Disagree (1) | Disagree (2) | Neither Agree, or Disagree (3) | Agree  (4) | Strongly Agree  (5) |
| 3. | **(The AFRICAT tool) seems applicable.** | Strong Disagree (1) | Disagree (2) | Neither Agree, or Disagree (3) | Agree  (4) | Strongly Agree  (5) |
| 4. | **(The AFRICAT tool) seems like a good match.** | Strong Disagree (1) | Disagree (2) | Neither Agree, or Disagree (3) | Agree  (4) | Strongly Agree  (5) |

| **Feasibility of Intervention Measure (FIM)** | | | | | | |
| --- | --- | --- | --- | --- | --- | --- |
| **1.** | **(The AFRICAT tool) seems implementable.** | Strong Disagree (1) | Disagree (2) | Neither Agree, or Disagree (3) | Agree  (4) | Strongly Agree  (5) |
| 2. | **(The AFRICAT tool) seems possible.** | Strong Disagree (1) | Disagree (2) | Neither Agree, or Disagree (3) | Agree  (4) | Strongly Agree  (5) |
| 3. | **(The AFRICAT tool) seems doable.** | Strong Disagree (1) | Disagree (2) | Neither Agree, or Disagree (3) | Agree  (4) | Strongly Agree  (5) |
| 4. | **(The AFRICAT tool) seems easy to use.** | Strong Disagree (1) | Disagree (2) | Neither Agree, or Disagree (3) | Agree  (4) | Strongly Agree  (5) |

## Appendix 5: Stakeholder Interview Guide

#### **1.** Defining the problem

In your view, what are the biggest mental health challenges that young people in your (family/community/ country) face?

- Which challenges do you notice most often?
- Are these challenges the same for boys and girls?

#### 2. Access to services

If a young person in your family or community needed mental health support, where would they go first (e.g., school, clinic, church, traditional healer, online)?

- How easy or challenging is it for young people to access mental health support?

#### 3. Barriers to care

What difficulties do families face when trying to access mental health support for adolescents?

- Is cost a problem?
- Does stigma play a role?
- Are there enough services nearby?
- Is quality of services a concern?
- What other concerns affect access to care?

#### 4. Early identification

How easy or difficult is it for parents and families/ teachers/ primary healthcare staff to recognise when a young person is struggling with their mental health?

- What signs do they usually notice?
- Are there signs that are often missed?
- Do young people ask for help? Why/ why not?

#### 5. Diagnosis

How do you feel about young people being given a mental health diagnosis?

- Do you think it is helpful, worrying, or both?
- How do parents/families react to a diagnosis?
- Do you think a diagnosis changes how a young person is treated?
- Are you aware of differences in the treatment of depression, anxiety and other common mental health conditions among adolescents?

#### 6. Assessment experience

Have adolescent in your care/ your child ever been asked to fill in a mental health questionnaire/ assessment? How did you feel about that experience?

- Was it helpful or uncomfortable?
- What would make it better?
- Do you think there could be value in regularly assessing young people’s mental health to identify who might benefit from treatment and see if they are getting better or worse?

#### 7. Online tools (general)

Would you feel comfortable about adolescents/ your child using a computer- or phone-based tool to check their mental health? Why or why not?

- What would make you trust/distrust the results?
- What are the potential benefits/ risks?

#### 8. Tool design – content

If adolescents in your care/your child used an online tool, what kind of information or feedback would you want it to give them (and you)?

- Should it give advice for parents/ healthcare workers/ teachers as well as children?
- Should it include referrals to services or contact numbers?
- Should it be directly linked to mental health awareness/ self-help materials?

#### 9. Tool design – questions

What types of questions would you be comfortable with adolescents in your care/your child answering in a mental health tool like AfriCAT?

- Which areas are most important?
- Are there questions you would not want included?
- Are there topics you feel are too sensitive?

#### 10. Tool design – features

What features would make you feel the AfriCAT tool is safe and supportive for young people to use (e.g., privacy settings, emergency contacts, referrals)?

- Which features would give you peace of mind?
- Which features would adolescents/your child/ you care most about?

#### 11. Perceptions of AfriCAT

The AfriCAT tool is designed to adapt the questions it asks based on how a young person has responded to previous questions. The idea is to ask *as few questions as possible* and *only the questions that are relevant to each person*, so that you can reach an assessment as quickly as possible

- How would you feel about adolescents in your care/your child using this type of tool?
- What are the potential benefits/ risks?

#### 12. Acceptability

Do you think young people/ parents and families/ teachers/ primary healthcare workers in your community would accept and trust a tool like AfriCAT? Why or why not?

- What might make them hesitant?
- What would build trust?

#### 13. Feasibility

Do you think it would be feasible to use a tool like AfriCAT to help adolescents in your community to get the right support?

- What are the opportunities for using the tool?
- What barriers would there be to using the tool?

#### 14. Safety & privacy

What concerns do you have about adolescents/ your child’s privacy, safety, or stigma if they used a mental health assessment tool like AfriCAT?

- What safeguards should be put in place?
- Who should have access to the results?
- Would your child feel safe using it?

#### 15. Implementation settings

Where do you think would be the best place for adolescents/ your child to use AfriCAT – at school, at a clinic, at home, or somewhere else? Why?

- Which setting feels safest?
- Which setting feels most feasible/ realistic?

#### 16. Integration into stepped-care models

If AfriCAT showed that an adolescent/ your child needed support, what would be the next best steps?

- Explore:
  - Information & supported self-care
  - Referrals to:
    - a lay or peer counsellor
    - a primary healthcare worker
    - a mental health specialist (psychologist/ psychiatrist)
  - Other community-based resources for adolescent mental health support (teachers/pastors/ traditional healers/sports or cultural clubs)
- When should care be “stepped-up” or stepped-down”?
- What would make referral/ coordination of care between providers easier?

#### 17. Oversight/responsibility

Who do you think should be responsible for making sure AfriCAT is safe and appropriate for adolescents to use (e.g., teachers, health workers, government/ NGOs)?

- Should this responsibility be local or national?
- Should parents/families have a role too?

#### 18. Cost & affordability

Would you be concerned about the cost of AfriCAT, or whether all young people could access it equally?

- Should it be free for all young people?
- Would cost stop people from using it?

#### 19. Equity & inclusion

Do you think all adolescents – including those in rural or low-income communities – would be able to access and benefit from AfriCAT?

- What barriers might some (e.g. rural) communities face?
- What would make it more inclusive?

#### 20. Opportunities

What positive changes do you think could come from having a mental health assessment tool like AfriCAT available for young people?

- Could it help young people and families talk about mental health?
- Could it make support easier to find?
- Could it contribute to addressing stigma towards mental health?

#### 21. Challenges

What challenges do you think young people/ families might face if they tried to use a mental health assessment tool like AfriCAT?

- Would technology be a challenge?
- Would stigma be a challenge?
- Would access to support for young people identified as having mental health challenges be a challenge?

#### 22. Evidence needs

What would you need to see or know before trusting adolescents in your care/your child to use a mental health assessment tool like AfriCAT over?

- Would research evidence convince you?
- Would hearing from other people/families help?
- Would hearing from mental health providers/ specialists help?

#### 23. Cultural relevance

How should AfriCAT consider cultural values, languages, and family traditions in its design?

- Should it be available in local languages?
- How should it address traditional beliefs?

#### 24. Youth participation

How do you think young people themselves should be involved in shaping and improving AfriCAT?

- Should young people be part of designing the tool?
- Should young people test the tool?

#### 25. Systems integration

How could AfriCAT connect with schools, clinics, or community programs to strengthen support for adolescents and families?

- Should schools play a bigger role?
- Should health services be linked directly?
- Should AfriCAT work with community-based/ non-governmental organisations?

## Appendix 6. Distress Protocol

This document outlines the standard operating procedures (SOPs) for identifying, responding to, mitigating and documenting risks/harms and adverse events occurring during the AfriCAT Study. All investigators interacting with adolescents will be trained on the contents of this protocol and the be responsible for implementing these procedures, with oversight provided by the Principal Investigators.

1. **Anticipated Risks**

Based on the criteria outlined by the HREC risk-assessment table, it is expected that study participants meet the criteria to be classified as members of a vulnerable group, as they:

- Under the age of 18 years
- Self-Identify as having a mental health condition, including depression or anxiety

The AfriCAT lived experience engagement activities will consist of two engagmenent workshops, which include focus group discussions and self-administered surveys.

It is anticipated that participants may be exposed to the following risks related to participation in the study:

- **Psychological Distress:** Psychological distress includes feelings of distress (worry, agitation, irritability, depressed mood) arising during or after participation in study activities. Psychological distress could arise from participating in focus group discussions or interviews covering topics of mental health, including lived experiences, mental health symptoms, access to mental health services, treatments and diagnosis, and mental health stigma. Whilst we will not enquire directly, participants may self-report personal experiences of trauma, stigmatization, self-harm, suicidal ideation/attempts, or other personal experiences that have the potential to cause psychological distress for themselves or other participants.
- **Stigmatization and or profiling:** Risks of stigma and profiling include self-stigma and stigma associated with being identified by others as having a mental health condition. Inclusion in the study as an adolescent who identifies as having mental health conditions such as depression or anxiety may expose adolescents to stigma and profiling.
- **Breach of Confidentiality:** Whilst investigators will endeavour to maintain participants anonymity and confidentiality, focus group discussions will be attended by other adolescents with lived experience hence it is not possible to maintain anonymity within the group.
- **Coercion:** Adolescents with lived experience may feel compelled to participate in the study or disclose lived experiences.

Additional anticipated risks/ harms which may arise during the course of the study/ be voluntarily disclosed by study participants, but not be related to participation in the study, may include:

- **Severe acute psychological distress**, including signs or symptoms of severe depression, anxiety, panic attacks, mania or psychosis
- **Suicidal ideation or self-harm with immediate risk of harm to self**
- C**urrent or on-going physical, sexual or emotional abuse or neglect**

2. **Risk Mitigation**

The investigators will take proactive steps to mitigate potential harms that could arise from the AfriCAT Study, including through participation in lived experience and stakeholder engagement activities.

**General risk mitigation strategies:**

- Lived experience engagement activities will be conducted by **suitably qualified investigators** with experience working with adolescents and facilitating focus group discussion and in-depth interviews, and overseen by a site-based Principal Investigator (site PI).
- A **Risk Management Team** composed of clinically qualified investigators with experience working with adolescents with mental health challenges, will oversee risk management.
- Each of the study sites have **existing networks and referral pathways** to primary healthcare providers, non-governmental organisations, and mental health and psychosocial support providers
- During early engagements with relevant stakeholders, contact information will be obtained for **emergency telephonic mental health services** and a registered counsellor/ clinical psychologist to be included in the study information sheets and consent/assent forms for each of the study sites.

**Risks of psychological distress, stigmatization and profiling, breaches of confidentiality and/or coercion will be mitigated by:**

- Ensuring fully informed consent or assent and parental/guardian consent (if under 18 years) with care to explain potential risks and to avoid coercion
- Avoiding direct enquiry regarding personal experiences of particularly sensitive topics such as trauma, self-harm, and suicidal ideation/attempts
- Encouraging adolescents to only share what they feel comfortable to share in the interviews and focus group discussions
- Identifying and reinforcing existing coping mechanisms and access to formal and informal mental health and psychosocial support services
- Providing contact numbers for the principal investigator and 24-hour toll-free national helplines

3. **Risk Identification**

Risks/ harms may be self-reported by participants to investigators during/ or after participation in interviews or focus group discussions. Investigators will be trained to identify harms and create a safe environment for adolescents to be comfortable to disclose harms if desired.

4. **Risk Response**

Risk responses will be standardised according to the Distress Protocol (this document). Risk responses reflect best practices and/or an enhanced standard of care in the settings in which the studies are being conducted, and are supported by agreements between the investigators and local service providers.

**4.1. Response to Severe Risks/Harms**

It is anticipated that investigators may become aware of the following severe risks/ harms:

1. **Severe acute psychological distress** requiring timely clinical assessment and management (including signs or symptoms of severe depression, severe anxiety, panic attacks, mania or psychosis)
2. **Suicidal ideation or self-harm with immediate risk of harm to self/others** as determined by a suitably qualified professional
3. **Current or on-going physical, sexual or emotional abuse or neglect,** including of a minor.

The following response should be implemented in the event of of severe risks/ harms:

- Provide immediate comfort to the participant by adhering to the following guidelines:
  - Remain calm and establish immediate safety
  - Remove any immediate hazards or call for help if required
  - Acknowledge the participants' report and reinforce their help-seeking behaviour
  - Assure them that you will uphold their safety, privacy, and work together to find a solution
  - Refrain from making any personal/ value judgments
  - Avoid making false reassurances such as ‘everything will be all right now’
- Ask the participant if there is a trusted family member or friend that you/ they could contact to be with them and help them access care
- Collaboratively identify resources/ support services available to the participant and agree on a safety/ management plan
- Inform the participant that you will need to complete a Case Report Form and refer the case to the site PI/Principal Investigator
- Complete a Case Report Form with an assessment of the risk/harm including onset, nature, severity, other risk or protective factors. Describe initial response, and further management/ safety plan.
- Obtain contact details for the participant [telephone number(s), physical address], as well as the contact details of a trusted family member or friend if the participant identifies one easily and would like this family member or friend to be a support.
- Immediately refer the case to the site PI/ Principal Investigator to obtain their input on the initial steps taken and further management steps required.
- If the harm meets criteria as a “severe adverse event” the site PI/Principal Investigator will report the harm to the ethics committee within 7 days of becoming aware of the event (see section 5 below).
- The site PI will be responsible for overseeing mandatory reporting of child abuse to relevant authorities in line with national laws and professional codes of conduct

**4.2. Psychological distress in the context of focus group discussion/ interviews**

A minimum of two investigators (a facilitator and an observer) will be present for all focus group discussions. In the event that a participant discloses symptoms of acute psychological distress, or exhibits signs of acute psychological distress (e.g. crying, shaking/ trembling, collapse) investigators should:

- Pause the focus group discussion/ interview and offer the participant immediate support.
- In the context of focus group discussion, the observer should offer the acutely distressed participant to move away from the focus group to discuss the matter privately.
- Assess the psychological/emotional status of the participant by probing what their thoughts and feelings are
- If the participant indicates that they feel able to continue with the focus group/ interview, resume with the focus group/ interview.
- If not, excuse the participant from participating and encourage the participant to contact the counselling service provider indicated on the information sheet if they experience increased distress in following hours/days
- Follow the participant up with a courtesy call
- One distressed participant in the focus group might also cause other focus group participants to become distressed. Regardless of whether the distressed participant excuses themself, the facilitator will engage with the whole group, to ensure that other participants are not experiencing distress.
- The facilitator will then ask the rest of the focus group members whether they would like to continue with or reschedule the focus group session.
- If the other participants indicate that they would like to continue with the focus group, the facilitator will resume the focus group.
- If not, the facilitator will reschedule the focus group and encourage the participants to contact the counselling service provider indicated on the information sheet if any of them experience increased distress in hours/days following the focus group.
- Debriefing: After the focus group has ended the facilitator and observer will summarise the content to the participants and key issues around confidentiality and anonymity will be reiterated. Potential sensitive and problematic issues that were raised during the focus group discussion will be clarified and contextualised where it was not appropriate to do so at the time. Participants will then be invited to share their reactions to such matters. The investigator can stay in the room at the end of the focus group and make her or himself available to give individual participants an opportunity to share their reactions.

5. **Reporting of adverse events**

**Serious adverse events are defined as:**

Any untoward physical, psychological or social occurrence that:

- Results in death
- Is life-threatening, including but not limited to suicide attempts/ self-harm requiring urgent medical attention
- Results in inpatient hospitalisation or prolongation of existing hospitalisation of > 5 days due to physical illness or injury
- Results in persistent or significant disability/incapacity
- Results in immediate danger or risk of ongoing danger to the participant or other minors, including interpersonal violence, sexual assault

Serious adverse events will be documented by the investigator on a Case Report Form and referred to the site PI immediately. The Risk Management Team will review the case and support the site PI to implement an appropriate safety/ management plan. The site PI will send an anonymised Case Report Form of the severe adverse event to the chair of the ethics committee within 7 days of becoming aware of the event/ harm.

The Risk Management Team will use their clinical judgment to decide whether or not an adverse event is of sufficient severity to require the participant’s removal from the study. A participant may also voluntarily withdraw from the study due to what he or she perceives as an intolerable AE. If either of these occurs, the participant must undergo an end of study assessment and be given appropriate care under medical supervision until symptoms cease, or the condition becomes stable.
