# Supplementary material for: Measurement-Based Mental Health Care for Adolescents in Africa: Protocol for a Computerized Adaptive Test
Source: JMIR Res Protoc. 2026 Sep 8;15:e98369. doi: 10.2196/98369 (PMC13552835; doi:10.2196/98369)
Supplement: Supplementary file 2 [file resprot-v15-e98369-s002.docx]

Multimedia Appendix 2

# Funder peer-review reports from the Wellcome Trust/ APHRC Mental Health Data Prize Africa review process

**Reviewer 1**

This is a very intriguing and innovative proposal designed to improve screening test for depression and anxiety among youth to aid both research and clinical treatment. The proposal is very clearly written and presented and a pleasure to read. The research team has outstanding credentials and represent a diversity of disciplines and lived experience. The key hypothesis/outcome of the project will be to determine if the adaptive test, using new technology, will be better and more precise than the static traditional measures. The integration of data sources represent intriguing innovation. The adaptive test will essentially build on 4 existing data sets. A potential limitation not addressed in the proposal are any obvious biases or shortcomings of these existing data sets. This is an important omission as any biases already present may be exacerbated in the new adaptive test. This would be a very important consideration, especially since the team is referring to this new adaptive test as representative of "African" populations even though only two countries are represented. The project still has tremendous significance and potential contribution, but it is important to not overstate the reach and implications. Finally, as mentioned Kenya and South Africa are considered middle income countries with more developed infrastructure than some of their neighboring countries. As a reader I find myself asking what may be the uptake of this adapted test, so more details on the front end and planning for intentional engagement will ensure any investor that the application and technology will be used and considered beneficial where it is most needed.

**Reviewer 2**

The proposal aims to use data collected from several studies in Kenya and South Africa to develop a new questionnaire for mental health. This questionnaire will be digitised. The said studies in Kenya and South Africa used PHQ-9 and GAD-7.

The two challenges mentioned: challenges in the identification of depression and anxiety at facilities and lack of data on depression and anxiety are not necessarily addressed or solved by what is being proposed in the study. Creating a new questionnaire that adolescents use to self-evaluate may not solve the challenged of identification of mental health problems. This may result in many false positives and further complicate diagnosis. Similarly, the fact that there is less data on mental health may be somehow solved by making a questionnaire available online, but may not. Lots of bad quality data may also result from this.

The authors do not explain whether PHQ-9 or GAD-7 are suitable to be used for adolescents, are these suitable to be used by the general population? These instruments are used in clinics to monitor progress or response to treatment of patients. The comment that these are static tool is not convincing. The ways in which these are used may not be static.

What are the risks involved when a teenager who may go though a difficult time but not necessarily experiencing clinical depression takes this questionnaire and may even self-medicate for example?

The CAT tools are adaptive in the sense that they can change the order or the questions used. In that sense they are dynamic, but they are highly dependent on the initial questions, and may introduce more biases and inconsistencies in the data.

I do not think that the authors discus their data sufficiently. Are the pool of questions that the new tool would use coming only from the instruments used on the said studies? Is it that the order of questions would change? or that some questions would be left out? In essence what the authors seem to imply is that only the answers of a few of the questions in a questionnaire are indicative of someone's depression for example. Or they imply that a blend of the questionnaire would be used. Otherwise how would Objective 2 make sense?

I do not understand the meaning and use of data harmonisation of the data from all these multiple questionnaires. What is being harmonised?

The ethical issues that authors discuss are all about data use, none are the implications of the use of the tool. I do not think that the tool itself can be validated properly by the methodology proposed.

**Comments from MHDP-Africa Secretariat**

The proposal titled "Computerised Adaptive Testing for Depression and Anxiety amongst Adolescents in African Populations (AfriCAT-DA)" outlines a project designed to develop a scalable and efficient digital tool for diagnosing depression and anxiety among adolescents in Africa. Here is a detailed summary of the project, along with an analysis of its novelty and innovative contributions:

Problem Statement: Depression and anxiety are leading causes of disability, particularly among adolescents, who are at a critical developmental stage. In Africa, mental health disorders are significantly underdiagnosed and untreated, especially due to socioeconomic challenges, lack of mental health services, and stigma. African adolescents face unique adversities, including poverty, food insecurity, trauma, and exposure to HIV, further contributing to mental health issues.

Objectives:

Develop a Computerised Adaptive Test for depression and anxiety tailored for adolescents in African populations (AfriCAT-DA).

Assess the efficiency and precision of AfriCAT-DA compared to static tools like the PHQ-9 (Patient Health Questionnaire) and GAD-7 (Generalized Anxiety Disorder scale).

Methodology:

Data Collection and Harmonization: The study utilizes de-identified individual data from adolescent mental health surveys conducted in South Africa and Kenya. The data will be harmonized and used to develop the AfriCAT-DA algorithm, incorporating advanced statistical and machine learning techniques.

Development and Validation: A subset of the data will be used to develop the adaptive test, and the remaining data will be used for simulation studies to validate the tool's efficiency, diagnostic accuracy, and predictive capability.

Evaluation Metrics: The efficiency and accuracy of AfriCAT-DA will be assessed by comparing its sensitivity, specificity, and Area Under the Receiver Operating Curve (AUROC) with existing tools.

Key Outputs and Anticipated Outcomes:

AfriCAT-DA Tool: A scalable, computerised adaptive test designed to identify depression and anxiety in adolescents, tailored for African contexts.

Publications and Policy Briefs: Research publications and policy briefs will summarize the findings, inform policymakers, and guide future interventions.

Community Engagement: Workshops with adolescents, caregivers, educators, and health workers will ensure the tool is culturally appropriate and adapted for use in schools and primary healthcare settings.

Social Impact: Early detection and treatment of depression and anxiety will improve mental health outcomes, reduce stigma, and enhance overall well-being.

Team Composition: The project team includes experienced researchers and data scientists from South Africa, Kenya, and international institutions. The team is diverse, with representation from various fields, including mental health, digital innovation, biostatistics, and community engagement.

Scalability and Long-term Vision: The project has the potential for scalability, expanding beyond South Africa and Kenya to other African countries. The AfriCAT-DA tool could be integrated into school-based programs and healthcare settings, creating a sustainable model for mental health diagnosis and care. Future iterations could include other mental health conditions and broader population groups.

Tailoring to African Adolescents: The tool will be culturally and contextually adapted for African adolescents, a demographic often neglected in global mental health research. By addressing the unique mental health challenges faced by African adolescents, the project creates a solution that is directly relevant to the population.

Data-Driven Approach: The project employs advanced statistical models (multidimensional item response theory) and machine learning to improve the diagnostic precision of the AfriCAT-DA. The innovative use of data from existing mental health studies to develop this tool ensures that it is grounded in real-world evidence.

Potential for Integration with Digital and Stepped Care Models: The AfriCAT-DA tool can be integrated with digital health interventions and stepped-care approaches, providing a scalable solution for managing mental health disorders. This integration makes the tool more relevant for low-resource settings, where access to mental health professionals is limited.

Focus on Gender and Equity: The project explicitly addresses gender disparities in mental health by analyzing results disaggregated by gender and poverty indicators. This focus ensures that the tool is inclusive and addresses the specific needs of vulnerable populations.

Scalability and Broader Applications: The AfriCAT-DA tool is designed to be scalable, potentially expanding to cover a range of mental health conditions and populations beyond adolescents. It could be used in schools, primary healthcare settings, and even integrated into national health surveillance systems to improve mental health data collection and service delivery across Africa. However, It is not clear what they want to do with the tools, if they want to replace the previous tools and why the CAT tool will be better?

They mention data from Kenya which they still do not have access to

How will the data they have help them to develop the said CAT

The idea seems like it is all about digitizing the software so where is the novelty?

The questionnaire said it's not a psychiatric tool

The PHQ9 and GAD-7 data has in-depth details and are therefore able to address a lot of questions, How will they use the data from the survey to address what these tools have

The Team seems to have only one person from Kenya who is based in the UK
